# The Importance of Velocity Acceleration to Flow-Mediated Dilation

**DOI:** 10.1155/2012/589213

**Published:** 2012-01-19

**Authors:** Lee Stoner, Joanna M. Young, Simon Fryer, Manning J. Sabatier

**Affiliations:** ^1^School of Sport and Exercise, Massey University, Wellington, New Zealand; ^2^Lipid and Diabetes Research Group, Diabetes Research Institute, Christchurch Hospital, Christchurch, New Zealand; ^3^School of Sciences and Physical Education, University of Canterbury, Christchurch, New Zealand; ^4^Division of Biological Sciences, Clayton State University, Morrow, GA, USA

## Abstract

The validity of the flow-mediated dilation test has been questioned due to the lack of normalization to the primary stimulus, shear stress. Shear stress can be calculated using Poiseuille's law. However, little attention has been given to the most appropriate blood velocity parameter(s) for calculating shear stress. The pulsatile nature of blood flow exposes the endothelial cells to two distinct shear stimuli during the cardiac cycle: a large rate of change in shear at the onset of flow (velocity acceleration), followed by a steady component. The parameter typically entered into the Poiseuille's law equation to determine shear stress is time-averaged blood velocity, with no regard for flow pulsatility. This paper will discuss (1) the limitations of using Posieuille's law to estimate shear stress and (2) the importance of the velocity profile—with emphasis on velocity acceleration—to endothelial function and vascular tone.

## 1. Introduction

The pathological complications of atherosclerosis, namely, heart attacks and strokes, remain the leading cause of mortality in the Western world [1]. Preceding atherosclerosis is endothelial dysfunction [2–4]. The flow-mediated dilation (FMD) test has emerged as the noninvasive standard for assessing *in vivo* endothelial function [5]. Despite its potential, the validity of the FMD test has been questioned due to the lack of normalization to the primary stimulus, shear stress [6–10]. Fortunately, the ultrasound technology used to conduct the FMD test can also provide estimates of shear stress [11]. Typically, shear stress is estimated by employing a simplified mathematical model based on Poiseuille's law. More sophisticated approaches using magnetic resonance imaging are available, but are beyond the reach of most clinical studies since such techniques are not readily available, are too expensive, and are technically challenging and time consuming [12–14].

Little attention has been given to the most appropriate blood velocity parameter(s) for calculating shear stress. The pulsatile nature of blood flow exposes the endothelial cells to two distinct shear stimuli during the cardiac cycle: a large rate of change in shear at the onset of flow (velocity acceleration), followed by a steady shear component. *In vitro* studies suggest that these two distinct fluid stimuli regulate short- and long-term endothelial function via independent biomechanical pathways [15–17]. The parameter typically incorporated into the Poiseuille's law equation for shear stress is time-averaged blood velocity, that is, blood velocity averaged across the cardiac cycle, with no regard to flow pulsatility. This paper will discuss (1) the limitations of using Posieuille's law to estimate shear stress and (2) the importance of the velocity profile—with emphasis on velocity acceleration—to endothelial function and vascular tone.

## 2. Flow-Mediated Dilation

The FMD test is a noninvasive method of evaluating endothelial function. A number of authors have developed standardized guidelines for conducting this test [18–20], including a recent article by Thijssen and colleagues [21]. The standard FMD test, as first described by Celermajer et al. [22], places a pneumatic tourniquet forearm just below the elbow and distal to imaged brachial artery. The tourniquet is inflated to a suprasystolic blood pressure for 5 minutes. Rapid deflation of the tourniquet instigates increased blood flow (reactive hyperemia) to the oxygen starved forearm muscles, with a subsequent increase in flow through the upstream brachial artery. The flow-induced increase in *shear stress* results in vasodilation of the brachial artery. The magnitude of FMD, expressed as the percentage increase in diameter above rest, is used to represent endothelial health.

## 3. Shear Stress

**Figure 1:**
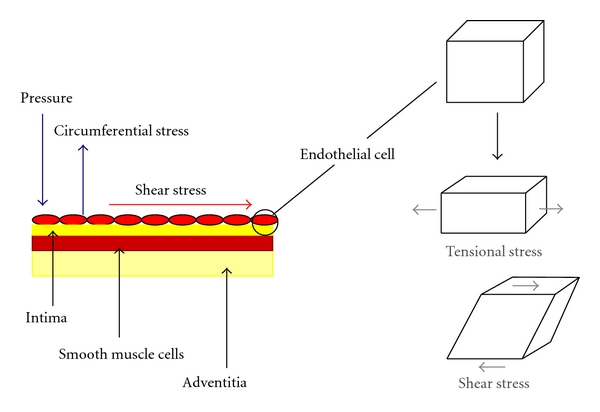
*Haemodynamic Stress.* Shear stress results in parallel deformation, as opposed to normal stress or force, which when applied to an object induces normal (direct) deformation.

**Figure 2:**
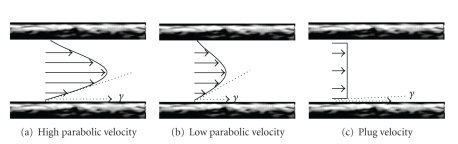
*Determination of Shear Rate (*γ*).* As fluid particles “travel” parallel to the vessel wall, their average velocity increases from a minimum at the wall to a maximum value at some distance from the wall, resulting in a gradient of velocities. The actual shear rate at the vessel wall is determined by shape of the velocity profile. Shear stress-induced deformation of the endothelial cells is detected by mechanoreceptors on the cell membrane.

Despite the term *flow*-mediated dilation, *shear stress* (see Figure 1) is the established stimulus for FMD [6, 7, 10, 23–28]. Shear stress is determined by red blood cells moving close to the endothelial cells. As the fluid particles “travel” parallel to the wall, their velocity increases from zero at the wall to a maximum value at some distance from the wall. This leads to the establishment of a gradient, which is defined as *shear stress *(Figure 2). Shear stress therefore acts at a tangent to the wall to create a frictional force at the surface of the endothelium. The endothelial cells are equipped with mechanosensors to detect this stress [29–38]. 

To maintain physiological levels of vessel wall shear stress, vascular tissues respond with acute adjustments in vascular tone through vasodilatation [39]. Vasodilation reflects alterations in the rate of production of endothelial-derived mediators, including nitric oxide (NO), prostacyclin (PGI_2_), and endothelial-derived hyperpolarizing factor (EDHF), which act locally to modulate vascular smooth muscle tone.

## 4. Shear Stress Mechanotransduction

The endothelium is a complex mechanical signal-transduction interface between the vessel wall and the flowing blood. Mechanotransduction is the interaction between shear stress-induced biomechanical forces and endothelial cell function. Exactly how these biomechanical forces are sensed by endothelial cells remains unclear. Two models of mechanotransduction have been demonstrated so far, a localized model and a decentralized model.

### 4.1. The Localized Model

**Figure 3:**
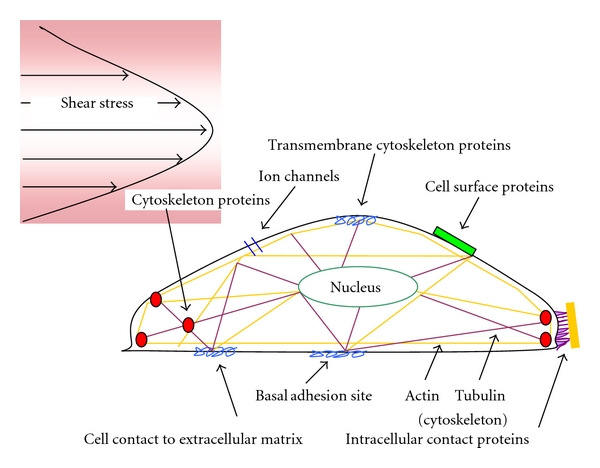
*Putative Mechanisms of Mechanotransduction*. The *localized* model for shear stress mechanotransduction assumes that shear stress sensors are located in the cell membrane. Ion channels are the proposed flow sensors. The decentralized *model* suggests that the mechanical forces acting on the luminal side of the endothelial cells are transmitted through the cytoskeleton to other sites in the cell. Integrins connected to the cytoskeleton have been related to this mechanism of mechanoreception.

The mechanoreceptor, like other receptors, is considered to be located in the cell membrane (Figure 3). Channels (i.e., K^+^, Na^+^, and Cl^−^) located in membrane respond to changes in shear stress. Because ion channel activation is one of the fastest known endothelial responses to flow, these ion channels are the proposed flow sensors [29–31, 33, 34, 40]. The flow-sensitive ion channel first identified in endothelial cells was an inward-rectifying K^+^ channel, whose activation leads to hyperpolarization of the cells membrane [35, 36]. The second type of flow-sensitive ion channel, more recently discovered, is an outward-rectifying Cl^−^ channel [37, 38]. The change in membrane potential, associated with the activation of these ion channels, alters the electrochemical gradient for Ca^2+^ transport across the endothelial cell membrane. This has been shown to provide a mechanism of direct interaction between flow-sensitive ion channels and Ca^2+^-dependent pathways [37, 38].

The Cl^−^ and K^+^ channels are activated independently [37, 38]. Activation of Cl^−^ channels leads to cell membrane depolarization; this follows the initial K^+^ channel-mediated hyperpolarization. The fact that hyperpolarization precedes depolarization, in spite of the larger electrochemical driving force for Cl^−^ than K^+^, suggests that flow-sensitive Cl^−^ channels attain maximal activation more slowly than flow-sensitive K^+^ channels. The notion that K^+^ channels respond to shear stress more rapidly than Cl^−^ channels is expected to be particularly relevant for situations where a time-varying shear stress may activate one or both channels depending on the time constant characterizing the changes in shear stress.

There is mounting evidence that flow-sensitive K^+^ and Cl^−^ channels play a central role in regulating overall endothelial responsiveness to flow [41–43]. This notion is supported by data demonstrating that interference with these candidate mechanosensors affects downstream gene and protein regulatory responses. For instance, pharmacological antagonists of flow sensitive K^+^ and Cl^−^ channels greatly attenuate or entirely abolish shear stress-induced release of cyclic guanosine monophosphate (cGMP) [41] and NO, downregulate endothelin-1 [42], and induce Na-K-Cl cotransport protein [43].

### 4.2. The Decentralised Model

This model suggests that the mechanical forces acting on the luminal side of endothelial cells are transmitted through the cytoskeleton to other sites within the cell [44]. The endothelial cell can be viewed as a membrane stretched over a frame composed of intermediate filaments and actin fibers which transverse the cells and end in adhesion complexes (Figure 3). Even under nonstimulated conditions, the entire endothelial cytoskeleton is maintained under tension, and in response to an externally applied stimulus intracellular tension is redistributed over the cytoskeleton network. These forces are especially sensed at the basal adhesion points, where the endothelial cell is attached to the extracellular matrix, cell junctions, and the nuclear membrane [30]. So it is conceivable that the application of a stressor activates signal transduction cascades without the need of a specific shear stress or stretch receptor. Integrins connected to the cytoskeleton have been related to this mechanism of mechanoreception [45]. 

## 5. Shear Stress Estimation

Clinical studies in humans, including FMD studies, typically estimate shear stress by employing a simplified mathematical model based on Poiseuille's law, where shear rate equals

(1) $$\text{Shear}\;\text{rate}\;\left(\gamma \right)=\;\frac{2\left(2+n\right)v}{d},$$

where *d* is the internal arterial diameter, *v* is time averaged mean blood velocity, and *n* represents the shape of the velocity profile. *For a fully developed parabolic profile, n is 2*.

 Poiseuille's law assumes that (1) the fluid (blood) is Newtonian; (2) blood flows through a rigid tube; (3) whole blood viscosity represents viscosity at the vessel wall and is linearly proportional to shear rate; (4) the velocity profile is parabolic; (5) mean blood velocity adequately defines the shear stimulus.

First, although blood is a non-Newtonian fluid at low shear rates (smaller than approx. 100 s^−1^) [46] *in vivo*, shear stress in large arteries, particularly at the endothelial surface, is generally considerably larger than this threshold value so that the effect of the non-Newtonian behavior does not appear to be pronounced. Second, blood vessels are distensible, meaning that wall shear rate may be ~30% less in a distensible artery as compared with a rigid tube [47].

Third, the magnitude of shear stress, to which the endothelial cell is subjected, is given by the product of the dynamic viscosity of blood and shear rate. Viscosity is an internal property of a fluid that offers resistance to flow. For Newtonian fluids, shear rate and viscosity are directly related. However, the relationship between shear rate and viscosity is nonlinear for non-Newtonian fluids. Human *in vivo* studies are usually limited to whole blood measurements of viscosity. These measurements overestimate the viscosity at the wall of the vessel. Less red blood cells travel along the artery wall, where, in addition to a thin layer of plasma, blood platelets are traveling [48]. Red blood cells tend to stream in the center of the vessel, resulting in higher viscosity in the center and thereby reducing the shear stress gradients at the vessel wall.

It is worth noting that shear stress assessments do not seem to result in conclusions different from shear rate assessments alone [49]. This may be explained by two factors: (1) sources of error from whole blood viscosity estimates and (2) blood viscosity exhibits low intrasubject variability [50], particularly among a healthy, homogenous group. Shear rate has been used as a surrogate measure of shear stress in a number of previous studies [49–53]. Nonetheless, the relationship between vascular homeostasis and blood viscosity is complex [54]. Further study is required to determine the influence of blood velocity on shear stress estimations, particularly for populations exhibiting cardiovascular risk factors known to effect blood viscosity.

Fourth, in arteries, the velocity profile will not develop to a full parabola as a consequence of flow unsteadiness and short vessel entrance lengths. In both arteries and arterioles, the velocity profiles are actually flattened parabolas ([13], see Figure  2B). In the common carotid artery, mean wall shear stress is underestimated by a factor of 2 when assuming a parabolic velocity profile [55]. In the brachial artery, the underestimation is less pronounced, likely due to a more parabolic velocity profile in this artery, that is, *n* (velocity profile) is closer to 2 ([55], see Figure  2A). However, this may only be true for resting conditions; occurrence of flow turbulence is possible during reactive hyperemia [56].

Fifth, for a given mean blood velocity, the flow profile can vary dramatically due to the pulsatile nature of circulation [57–59]. Blood flow pulsatility results in endothelial cells being exposed to two distinct shear stimuli during the cardiac cycle: a large rate of change (velocity acceleration) in shear at the onset of flow, followed by steady shear. *In vitro* studies suggest that these two distinct stimuli regulate short- and long-term endothelial function via independent biomechanical pathways [60–67]. Mean blood velocity is therefore unlikely to characterize the shear stimulus, particularly during hyperemic conditions.

## 6. The Importance of the Velocity Profile to Shear Stress Mechanotransduction

The earliest studies investigating the effects of shear stress on endothelial function did so by assessing endothelial cell responses to high versus low shear stress. This was until Davies et al. [68], in 1986, provided evidence that the time-averaged shear stress alone could not explain the pathological behavior of endothelial cells exposed to complex flow patterns. Subsequent studies [57, 66, 69–75] have shown that vascular endothelial cells respond not only to the time-averaged shear stress, but respond differently to different patterns of flow.

**Figure 4:**
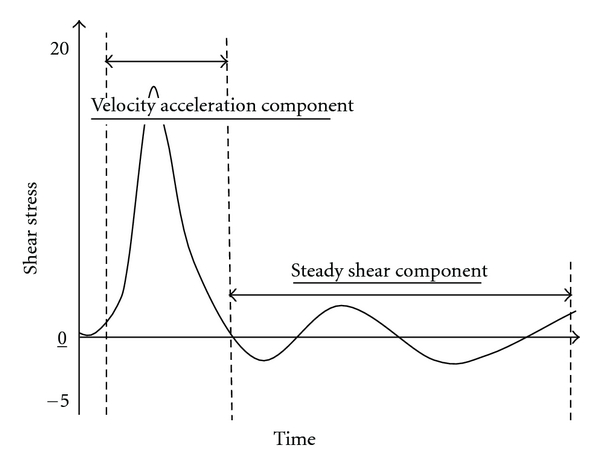
*Acceleration and Steady Shear Components*. The normal brachial arterial signal is *triphasic*, corresponding to the (1) rapid blood flow during systole, resulting in velocity acceleration, (2) initial reversal of blood flow in diastole, and (3) gradual return of forward flow during late diastole, resulting in steady shear component.

The cyclic nature of the beating heart creates pulsatile flow conditions in all arteries. The heart ejects blood during systole and fills during diastole. These cyclic conditions create relatively simple *monophasic* flow pulses in the upper region of the aorta [76]. However, pressure and flow characteristics are substantially altered as blood circulates through the arterial tree.  Figure 4 shows an example of a typical brachial artery blood velocity profile. The normal brachial arterial signal is *triphasic*, corresponding to (1) rapid blood flow during systole, (2) initial reversal of blood flow in diastole, and (3) gradual return of forward flow during late diastole.

The blood flow profile in the aorta is predominately governed by the force of blood ejected from the heart [77]. However, in the periphery, the blood flow profile becomes more complex as a result of the energy transfer between the heart and arteries. The heart generates forward-traveling wave energy that propagates through the arteries to maintain tissue and organ perfusion for metabolic homeostasis. An individual forward-traveling waveform, generated by the heart at the beginning of systole, initiates flow and increases pressure in the arteries. Although most of the wave energy in this initial compression wave travels distally into smaller arteries, some is reflected back towards the heart at sites of impedance mismatch. Interactions between forward- and backward-traveling waves result in complex blood flow patterns. Wave reflections result from arterial geometry, arterial wall compliance, and downstream resistance created by resistance arteries [57, 59].

Complex flow characteristics have a profound impact on the shear stress distribution to which vascular endothelial cells are exposed. While human *in vivo* studies typically describe shear stress as a mean construct, numerous secondary phenomena associated with flow, including pulsatile flow, retrograde flow, and flow turbulence, can influence the regulation of endothelial cells [23, 60, 62, 78–80]. 

### 6.1. Velocity Acceleration and Endothelial Function

**Figure 5:**
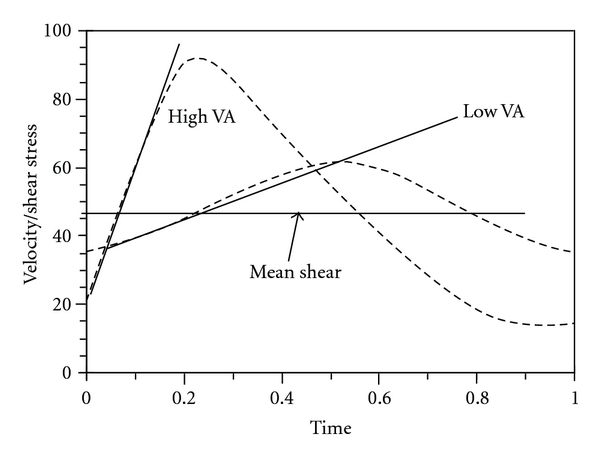
*High versus Low Velocity Acceleration (VA).* The horizontal line denotes identical mean shear stress for both VA rates.

The pulsatile nature of blood flow exposes the endothelial cells to two distinct shear stimuli during the cardiac cycle: a large rate of change in shear at the onset of flow (velocity acceleration), followed by a steady shear component (Figure 4). *In vitro* studies suggest that these two distinct fluid stimuli (velocity acceleration versus steady velocity) regulate short- and long-term endothelial function via independent biomechanical pathways [60–67]. For a given mean blood velocity, or shear stress, velocity acceleration can vary quite substantially (Figure 5). Studies have shown that the rate of velocity acceleration can affect the progression of atherosclerosis [60–62, 67, 81–85], endothelial cell function [62, 67, 86], mechanotransduction [63–65, 83, 87], calcium kinetics [88–91], and vascular tone [15–17, 92–94].

### 6.2. Velocity Acceleration and Flow-Mediated Dilation

The endothelium mediates flow-mediated vasodilation by altering the release of numerous factors, including NO, PGI_2_ and EDHF factor. The development of sophisticated *in vitro* flow models has allowed the effects of velocity acceleration on cultured endothelial cell function to be studied. Notably, the release of NO and PGI_2_, from cultured endothelial cells has been directly related to the rate of velocity acceleration [15, 16, 92, 93]. The rate of velocity acceleration has also been directly related to vasodilation of isolated cremaster arterioles [94].

**Figure 6:**
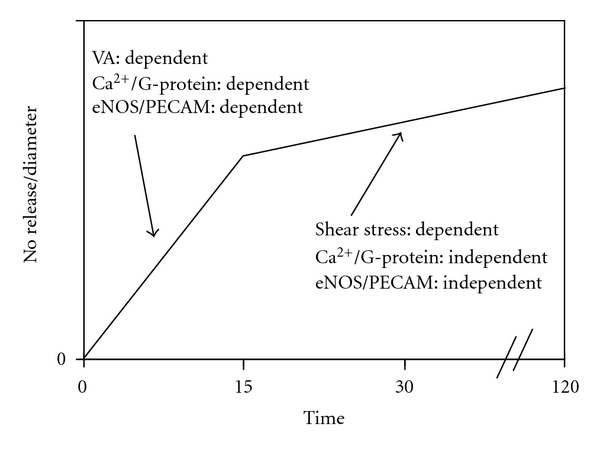
*Velocity Acceleration versus Shear Stress-Dependent Dilation*. Velocity acceleration (VA) and mean shear stress appear to regulate nitric oxide production via distinct pathways. Ca^+^: calcium; eNOS: endothelial nitric oxide synthase; PECAM: platelet/endothelial cell adhesion molecule.

The production rate of NO and PGI_2_ following flow onset exhibits a biphasic response, an initial transient burst followed by a slower release to a constant rate [15, 16, 92]. Separate studies from the same group [15, 16] demonstrated that the initial burst of NO is dependent on velocity acceleration but not the shear stress magnitude; the subsequent constant NO production is dependent on shear stress magnitude (Figure 6). The initial burst of NO was found to be Ca^2+^ and G-protein dependent (i.e., “localized” mechanotransduction). In contrast, the subsequent constant NO production was found to be Ca^2+^ and G-protein independent (i.e., “decentralized” mechanotransduction). A more recent study found that the initial velocity acceleration-dependent signal for NO release requires platelet/endothelial cell adhesion molecule-1 (PECAM-1) [17]. PECAM-1, which acts as an intracellular bridge between the two plasma membranes of neighboring cells, is complexed with endothelial nitric oxide synthase (eNOS) at the cell-cell junction. Velocity acceleration is thought to deform the endothelial cell plasma membrane and activate PCAM-1 [95]. The velocity acceleration-dependent burst is consistent with previous *in vitro* observations [96, 97] for flow-mediated release of PGI_2_ and for the stimulation of NO release by Ca^2+^ mobilizing agonists [98]. Similar findings have been observed in isolated perfused vessels exposed to acute changes in flow [41, 94, 99].

Recently, we studied the effect of velocity acceleration on FMD in a group of 14 healthy, young, male subjects [100]. FMD was measured prior to, and following, increases in velocity acceleration. Velocity acceleration was increased by inflating a tourniquet to 40 mmHg around the forearm. We found a 14% increase in velocity acceleration-attenuated FMD by 11%. This finding suggests that mean blood velocity alone may not adequately characterize the shear stimulus. Attention to secondary flow phenomena may be particularly important when comparing groups with known secondary flow abnormalities.

### 6.3. Potential Limitations of *In Vitro* Studies

The relevance of findings garnered from *in vitro* studies bear two important limitations. Firstly, endothelial cells from different tissue beds have been shown to exhibit different responses for a given flow paradigm [101]. For instance, it has been shown that different tissue beds exhibit different optimal flow frequencies for proliferation, eNOS activity, and PGI_2_ secretion [101]. Secondly, most culture studies have investigated endothelial gene regulation in response to a single type of stimulus (continuous laminar shear stress). Therefore, although these simple *in vitro* models have yielded valuable information, they fail to mimic the complexities of the *in vivo* environment. The applicability of these findings to humans remains to be determined.

## 7. Velocity Acceleration and Vascular Health

The effects of velocity acceleration on the development of atherosclerosis have produced conflicting findings. Bao et al. [60] found that velocity acceleration upregulated the expression of putative atherogenic genes (monocyte chemoattractant protein-1 and platelet-derived growth factor-A) which are believed to participate in the early events of atherosclerosis [102, 103]. The same group found that velocity acceleration upregulates endothelial cell proliferation [62]. However, a more recent study by Hsiai et al. [81] found that pulsatile flow actually downregulated the expression of monocyte chemoattractant protein-1 and reduced monocyte binding to lipid-oxidized endothelial cells. Furthermore, they found that the effects were more exaggerated for pulsatile flow with high velocity acceleration versus pulsatile flow with low velocity acceleration, even though mean shear stress was equivalent (50 dyne/cm^2^).

Apart from the use of different endothelial cell cultures (bovine aortic [60] versus human umbilical [81]), a fundamental difference between the two aforementioned studies lies in the flow paradigms utilized. Bao et al. [60] evoked a single flow impulse (abrupt 0 to 16 dyne/cm^2^ sustained for 3 seconds), whereas Hsiai et al. [81] evoked pulsatile flow with a mean shear stress of 50 dyne/cm^2^. Notably, the Hsiai et al. [81] flow model did not permit shear stress to return to zero between flow impulses, thereby, inducing a steady flow component. However, when Hsiai et al. [81] produced an oscillating flow profile (0 ± 5 dyne/cm^2^), which induced high velocity acceleration but had a mean shear stress of 0 dyne/cm^2^ and was devoid of a steady flow component, monocyte chemoattractant protein-1 expression was upregulated and monocyte binding was increased. This is consistent with the notion that, while endothelial cells derive directional cues from the flow direction or velocity, a certain persistence of the stimulus is required [86, 89]. Taken together, these findings suggest that endothelial cells are regulated by a complex interplay between steady flow/shear stress and velocity acceleration.

*In vivo*, flow is pulsatile everywhere in the arterial system, but in most places there is a large steady component. However, at sites where flow oscillates with a low steady component, atherosclerotic lesions are known to occur. Again, this is consistent with the notion that while endothelial cells derive directional cues from the flow direction or velocity acceleration, a certain persistence of the stimulus is required [86, 89]. Steady shear stress results in the continuous upregulation of antiatherogenic genes (manganese superoxide dismutase, cyclooxygenase-2, eNOS) [104]. Steady shear also promotes endothelial cell release of various bioactive substances that may be involved in the regulation of atherogenic genes, such as NO [105, 106]. These findings suggest that the production of atherogenic genes by endothelial cells is regulated by the complex interaction between velocity acceleration and steady flow components. 

### 7.1. Determinants of Velocity Acceleration

Velocity acceleration is altered by diseased states. Decreased velocity acceleration is seen with ventricular ischemia [107], acute myocardial infarction [108], and stenoses [109]; increased velocity acceleration occurs with hypertension [110], hyperthyroidism [111], bypass grafts [112], and obstruction of the lumen [113]. Velocity acceleration is also decreased by aging [110] and increased with physical activity [114–116] and vascular resistance [110, 117, 118]. Inadequate definition of the shear stimulus may hinder comparisons between the aforementioned patient groups, who exhibit differing rates of velocity acceleration even though mean blood velocities may be comparable. Therapies that affect velocity acceleration may also complicate longitudinal observations on patient groups.

## 8. Calculating Shear Stress

Future studies using the FMD test should *consider* both time-averaged mean blood velocity and secondary flow parameters, particularly when making between-group comparisons. Emphasis is placed on the word *consider* since the FMD test should not be normalized to shear stress using conventional approaches. A number of studies have normalized the FMD response through dividing FMD by the shear stimulus or using an analysis of covariance (ANCOVA) approach [49, 119–123]. However, when using a General Linear Model (GLM), the following assumptions must hold true: (1) there must be at least a moderate correlation between the two variables (i.e., shear and FMD); (2) the relationship between shear and diameter must be linear; (3) the intercept for the regression slope must be zero; (4) variance must be similar between groups; (5) data must be normally distributed [121, 124]. A recent study found that that all assumptions for reliable use of shear-diameter ratios were violated [122].

The FMD response (i.e., change in diameter) can be normalized to the shear stimuli using hierarchical linear modeling (HLM) [125]. The advantage of HLM is that it allows the researcher to look at hierarchically structured data and interpret results without ignoring these structures. This is accomplished in HLM by including a complex random subject effect which can appropriately account for correlations among the data. This approach models different patterns of growth trajectories by allowing for the intercepts (initial diameter) and slopes (shear rate diameter) to randomly vary. A third level may also be specified: the specification of groups (e.g., to delineate differences in endothelial function) and/or the specification of an intervention or a modifiable risk factor such as smoking. This approach has previously been used to compare upper versus lower extremity arterial health in persons with spinal cord injury (SCI) [126], to assess improvements in arterial health following electrical stimulation-evoked resistance exercise therapy in persons with SCI [127], and to assess the effects of occasional cigarette smoking on arterial health [28]. The disadvantage of this approach is that multiple stimuli (preferably ranging from minimal to maximal shear stimuli) are required to generate a reliable shear-diameter slope; this results in lengthened testing time and potentially makes it more difficult to ascertain the mechanism(s) resulting in dilation.

## 9. Conclusions

Pulsatile flow, present throughout the arterial tree, results in endothelial cells being exposed to two distinct shear stimuli during the cardiac cycle: a large rate of change (velocity acceleration) in shear at the onset of flow, followed by relatively steady shear.* In vitro* studies suggest that these two distinct fluid stimuli (velocity acceleration versus steady shear) regulate short- and long-term endothelial function via independent biomechanical pathways. Studies have shown that the rate of velocity acceleration can affect mechanotransduction, vascular tone, and atherosclerosis. Velocity acceleration may be altered in a number of diseased states, as well as by aging and physical activity. Velocity acceleration may be an important independent variable governing the shear stimulus and should be considered when comparing groups with known secondary flow abnormalities.
